# Antimicrobial Resistance among *Campylobacter* Strains, United States, 1997–2001

**DOI:** 10.3201/eid1006.030635

**Published:** 2004-06

**Authors:** Amita Gupta, Jennifer M. Nelson, Timothy J. Barrett, Robert V. Tauxe, Shannon P. Rossiter, Cindy R. Friedman, Kevin W. Joyce, Kirk E. Smith, Timothy F. Jones, Marguerite A. Hawkins, Beletshachew Shiferaw, James L. Beebe, Duc J. Vugia, Terry Rabatsky-Ehr, James A. Benson, Timothy P. Root, Frederick J. Angulo, for the NARMS Working Group

**Affiliations:** * Centers for Disease Control and Prevention, Atlanta, Georgia, USA,; † Minnesota Department of Health, Minneapolis, Minnesota, USA;; ‡ Tennessee Department of Health, Nashville, Tennessee, USA;; § Maryland Department of Health and Mental Hygiene, Baltimore, Maryland, USA;; ¶ Oregon Department of Human Services, Portland, Oregon, USA;; # Colorado Department of Public Health & Environment, Denver, Colorado, USA;; ** California Department of Health Services, Berkeley, California, USA;; †† Connecticut Department of Public Health, Hartford, Connecticut, USA;; ‡‡ Georgia Department of Human Services, Atlanta, Georgia, USA;; §§ New York State Department of Health, Albany, New York, USA

**Keywords:** *Campylobacter*, United States, antimicrobial resistance, humans, poultry, fluoroquinolone, macrolide

## Abstract

We summarize antimicrobial resistance surveillance data in human and chicken isolates of *Campylobacter*. Isolates were from a sentinel county study from 1989 through 1990 and from nine state health departments participating in National Antimicrobial Resistance Monitoring System for enteric bacteria (NARMS) from 1997 through 2001. None of the 297 *C. jejuni* or *C. coli* isolates tested from 1989 through 1990 was ciprofloxacin-resistant. From 1997 through 2001, a total of 1,553 human *Campylobacter* isolates were characterized: 1,471 (95%) were *C. jejuni*, 63 (4%) were *C. coli*, and 19 (1%) were other *Campylobacter* species. The prevalence of ciprofloxacin-resistant *Campylobacter* was 13% (28 of 217) in 1997 and 19% (75 of 384) in 2001; erythromycin resistance was 2% (4 of 217) in 1997 and 2% (8 of 384) in 2001. Ciprofloxacin-resistant *Campylobacter* was isolated from 10% of 180 chicken products purchased from grocery stores in three states in 1999. Ciprofloxacin resistance has emerged among *Campylobacter* since 1990 and has increased in prevalence since 1997.

*Campylobacter* is the most common cause of bacterial gastroenteritis in the United States, causing an estimated 2.4 million human infections annually (*1*). Diagnosed infections have declined in recent years. In 2001, FoodNet surveillance identified 13.4 diagnosed *Campylobacter* infections per 100,000 persons (*2*). Approximately 95% of diagnosed *Campylobacter* infections are due to *C. jejuni* (*3*). Although most *Campylobacter* infections cause an acute, self-limited illness characterized by diarrhea, fever, and abdominal cramps, severe infections do occur (*4*). Antimicrobial treatment can shorten the duration of illness and may be life-saving in invasive infections (*5**–**7*). Fluoroquinolones (e.g., ciprofloxacin) are often prescribed empirically for the treatment of gastroenteritis and for *Campylobacter* infections in adults (*6**,**8*). Quinolones (e.g., nalidixic acid), although now seldom used for treatment in the United States, are frequently used to screen for fluoroquinolone resistance because of the close correlation between quinolone and fluoroquinolone resistance among *Campylobacter*. Macrolides, such as erythromycin, are also prescribed to treat *Campylobacter* infections (*4**,**9*).

Fluoroquinolone-resistant *Campylobacter* infections in humans were first detected in Europe in the late 1980s (*10**–**12*). Subsequently, an increasing proportion of *Campylobacter* isolates around the world have been found to be fluoroquinolone-resistant (*13*). Studies in the United States, Europe, and New Zealand have identified poultry as a principal source of *Campylobacter* infection (*14**–**16*). Quinolones have been available in human medicine since the mid-1960s, and the first fluoroquinolone (ciprofloxacin) was approved for use in humans in 1986. Two fluoroquinolones, sarafloxacin and enrofloxacin, were approved for use in poultry by the U.S. Food and Drug Administration (FDA) in 1995 and 1996, respectively (*17*). These fluoroquinolones were the first ones approved in food animals; subsequently, other fluoroquinolones have been approved for veterinary use but not for use in poultry (*18*).

To investigate the epidemiology of fluoroquinolone-resistant *Campylobacter* in the United States, we reviewed national surveillance data to determine the prevalence of antimicrobial resistance, particularly ciprofloxacin resistance, among *Campylobacter* isolates; conducted a case-control study to determine the proportion of ciprofloxacin-resistant infections that were domestically acquired; and performed a retail survey to determine the prevalence of ciprofloxacin-resistant *Campylobacter* contaminating chicken products sold in selected supermarkets.

## Methods

### National Surveillance for Resistance in *Campylobacter*

#### 1989–1990 Sentinel County Study

From 1989 to 1990, a national county-based survey of antimicrobial susceptibility among *Campylobacter* isolates was conducted. Sentinel clinical laboratories in 19 counties participated. The methods of this survey are described elsewhere (*19**,**20*). Briefly, the first five sporadic *Campylobacter* isolates identified each month were forwarded to the Centers for Disease Control and Prevention (CDC). Patients with *Campylobacter* infection were interviewed with a standard questionnaire, which included information about clinical illness and exposures (i.e., food, animal, and foreign travel) during the 2 weeks before illness onset. Isolates were determined to be *Campylobacter* by dark-field microscopic examination and hippurate hydrolysis (*20**–**22*). Hippurate-positive isolates were considered *C. jejuni*. All isolates with questionnaires received during the first 4 months of the study underwent susceptibility testing. Because of a shortage of reagents, a random sample of 50% of isolates with completed questionnaires received during the last 8 months of the study was further characterized. Isolates were tested for susceptibility to azithromycin, chloramphenicol, ciprofloxacin, clindamycin, erythromycin, gentamicin, nalidixic acid, and tetracycline by using broth microdilution methods (*19*). In 2003, a retrospective analysis of the hippurate-negative isolates was completed; these isolates were speciated by using methods described below.

#### National Antimicrobial Resistance Monitoring System (NARMS), 1997–2001

NARMS for enteric bacteria is a collaboration between CDC, the Food and Drug Administration, and state and local health departments. The system monitors patterns of antimicrobial drug resistance. NARMS methods are described in detail elsewhere (*23*).

In brief, isolates were tested for viability, confirmed as *Campylobacter*, and identified to the species level by using the hippurate hydrolysis test according to published methods (*21**,**22*). Hippurate-negative *Campylobacter* in which the hippuricase gene could be detected by polymerase chain reaction (PCR) were identified as *C. jejuni* (*24*). Isolates that tested negative for the hippuricase gene but positive for a *C. coli*-specific ceuE sequence were identified as *C. coli* (*25*). Isolates that could not be identified as either *C. jejuni* or *C. coli* by these PCR assays were referred to the National *Campylobacter* Reference Laboratory at CDC for identification with genotypic (e.g., 16S rRNA sequencing) and phenotypic methods (*21*).

Isolates were tested with the E-test system (AB BIODISK, Solna, Sweden) to determine MICs for six antimicrobial agents: chloramphenicol, ciprofloxacin, clindamycin, erythromycin, nalidixic acid, and tetracycline. Beginning in 1998, azithromycin and gentamicin were also included. When available, National Committee for Clinical Laboratory Standards interpretive criteria for *Enterobacteriaceae* MICs were used; ciprofloxacin resistance was defined as MIC >4 µg/mL, and erythromycin resistance was defined as MIC >8 µg/mL (*26*). Multidrug resistance was defined as resistance to two or more of the original six antimicrobial agents.

We used a multivariable logistic regression model to assess changes in the proportion of isolates with antimicrobial drug resistance from 1997 through 2001 because the population under surveillance more than doubled from 1997 to 2001, and substantial site-to-site variation in prevalence of antimicrobial drug resistance was identified (i.e., uncertainty was found in the denominators for calculating rates). The model was for antimicrobial drug resistance as a function of year and included main effects adjustments for age categories and site-to-site variation in prevalence. Within the available data, site by year interaction was not a significant factor but because the catchment areas expanded, the hypothesis of site by year interaction could not be fully tested.

#### 1997 Retrospective Case-Comparison Study

Using NARMS isolates, we conducted a retrospective case-comparison study in four NARMS sites (California, Connecticut, Georgia, and Oregon). Persons with ciprofloxacin-resistant (CipR) *Campylobacter* infection identified in 1997 were compared with persons in whom the diagnosis of ciprofloxacin-sensitive (CipS) *Campylobacter* infection was made that same year. We compared up to two CipS cases for each CipR case and matched cases by geographic site and date of stool specimen collection. All case-patients were interviewed by telephone, usually within 8 weeks of their illness onset, about demographics, clinical information, and exposures (e.g., antimicrobial drug use in the 4 weeks before illness onset, foreign travel, and consumption of poultry and raw milk in the 7 days before illness onset) with a standardized questionnaire.

#### 1999 Retail Survey

Three NARMS-participating state health departments (Georgia, Maryland, and Minnesota) participated in a survey of retail chicken products. From January to June 1999, each site purchased a convenience sample of 10 whole broiler chickens per month from supermarkets located within the state. State public health laboratories at each site tested the samples for *Campylobacter*. Carcass rinse samples were centrifuged, and pellets were incubated in enrichment broth and plated onto *Campylobacter* blood agar plates according to methods published elsewhere (*27*); neither media contained quinolone or macrolide antimicrobial agents. *Campylobacter* isolates were forwarded to CDC for species identification and antimicrobial susceptibility testing according to NARMS methods.

## Results

### National Surveillance

#### 1989–1990 Sentinel County Study

Two hundred ninety-eight patients were interviewed, and their *Campylobacter* isolates were tested. Of these isolates, 289 (97%) were *C. jejuni*, 8 (3%) were *C. coli*, and 1 (0.3%) was a *C. lari*. None were resistant to ciprofloxacin, and 3 (1%) of 294 were resistant to nalidixic acid (MIC > 32 µg/mL); 1 isolate was *C. lari*, which is inherently resistant to nalidixic acid (*28*), and 2 were *C. jejuni*. The *C. lari* isolate was resistant to ofloxacin (MIC = 8 µg/mL), intermediately resistant to norfloxacin (MIC = 8 µg/mL) but susceptible to ciprofloxacin (MIC = 2 µg/mL).^1^ The two nalidixic acid-resistant *C. jejuni* isolates were susceptible to ciprofloxacin (MIC = 0.5 µg/mL) and norfloxacin and ofloxacin (MIC < 2 µg/mL). The proportion of the isolates resistant to tetracycline was 42% (124/295). The resistant proportion for the other antimicrobial agents tested were as follows: erythromycin 3% (8/295), clindamycin 2% (6/295), azithromycin 2% (5/294), chloramphenicol 0% (0/295), and gentamicin 0% (0/295). Travel history was available for 296 patients with *Campylobacter* infection; 23 (8%) patients traveled outside of the United States in the week before illness onset. Of the persons with available information, 32 (11%) of 295 had taken an antimicrobial agent in the 30 days before illness onset, 46 (15%) of 298 were hospitalized, and 241 (81%) of 297 were treated with an antimicrobial agent for their illness. Among the 234 persons for whom treatment data were available, the most common agents used for treatment were erythromycin (62%), ciprofloxacin (19%), and trimethoprim-sulfamethoxazole (5%). Of the three patients with nalidixic acid-resistant infections, none traveled outside the United States, and none were treated with a quinolone or fluoroquinolone in the month before illness.

#### NARMS, 1997–2001

From 1997 to 2001, a total of 1,932 presumptive *Campylobacter* isolates were received at CDC through NARMS; 193 (10%) were excluded because they were not viable, 104 (5%) were not in accordance with the one-a-week sampling method, 39 (2%) were determined not to be *Campylobacter*, 22 (1%) were duplicates, and 21 (1%) were contaminated cultures. Of the 1,553 (80%) isolates further characterized and included in this analysis, 1,471 (95%) were *C. jejuni*, 63 (4%) were *C. coli*, 7 (0.4%) were *C. upsaliensis*, 5 (0.3%) were *C. fetus*, 2 (0.1%) were *C. lari*, and 5 (0.3%) were undetermined (i.e., determination by 16S study did not identify a species). Forty-five percent of case-patients were female; the median age was 33 years (range <1–96). Among 1,439 isolates with known source of specimen collection, 1,426 (99%) were from stool samples, and 13 (1%) were from blood samples. Among blood isolates, eight were *C. jejuni*, two were *C. fetus*, two were *C. upsaliensis*, and one was *C. lari*.

The results of susceptibility testing among *Campylobacter* isolates by species are shown in Table 1. Resistance to ciprofloxacin among all *Campylobacter* isolates was 13% in 1997 and 19% in 2001. Resistance to erythromycin among all *Campylobacter* isolates was 2% in 1997 and 2% in 2001. The results of antimicrobial susceptibility testing by year for isolates of the most common species, *C. jejuni*, are shown in Table 2.

**Table 1:** Antimicrobial resistance among *Campylobacter* isolates by species, National Antimicrobial Resistance Monitoring System 1997–2001

Antimicrobial agent	% resistant						
	*C. jejuni* (n = 1,471)	*C. coli* (n = 63)	*C. upsaliensis* (n = 7)	*C. lari* (n = 2)	*C. fetus* (n = 5)	Undetermined^a^ (n = 5 )	Total (n = 1,553)
Azithromycin^b^	2	9	0	0	0	0	2
Chloramphenicol	0.3	5	0	0	0	40	0.6
Ciprofloxacin	16	30^c^	14	0	0	0	16
Clindamycin	1	9	0	0	0	20	2
Erythromycin	2	8	0	0	0	20	2
Gentamicin^b^	0	2	0	0	0	0	0.1
Nalidixic acid	17	36^c^	14	100	80	20	18
Tetracycline	43	43	0	0	20	0	43

^a^Undetermined isolates were hippurate-negative *Campylobacter* that could not be further speciated with available polymerase chain reaction primers. ^b^For azithromycin and gentamicin, only 1,336 isolates were tested. ^c^Comparison of proportion of resistant *C. coli* to resistant *C. jejuni* was statistically significant for ciprofloxacin and nalidixic acid but not tetracycline (p < 0.01)

**Table 2:** Antimicrobial resistance among human *Campylobacter jejuni* strains, 1989–1990 and 1997–2001

Antimicrobial agent	% resistant						
	1989–1990 (n = 286)^a,b^	1997 (n = 209)	1998 (n = 297)	1999 (n = 294)	2000 (n = 306)	2001 (n = 365)	Total (n = 1,757)
Azithromycin^c^	1	–	1	3	2	2	1
Chloramphenicol	0	1	1	0.3	0	0	0.3
Ciprofloxacin	0	12	14	18	14	18	13
Clindamycin	1	1	1	1	1	2	1
Erythromycin	1	1	2	2	1	2	2
Gentamicin^c^	0	–	0	0	0	0	0
Nalidixic acid	1	13	16	20	16	19	14
Tetracycline	42	47	46	46	39	40	43

^a^1989–1990 U.S. sentinel county study used different sampling and laboratory methods (microbroth dilution testing) than NARMS (Etest). However, studies have concluded that broth microdilution and Etest give equivalent results for ciprofloxacin susceptibility testing of *Campylobacter* (*44*). ^b^In 1989–1990 U.S. county study, only 285 isolates were tested for azithromycin and nalidixic acid susceptibility. ^c^For azithromycin and gentamicin, only isolates received between 1998 and 2001 were tested (N = 1,262).

The prevalence of ciprofloxacin-resistant *Campylobacter* ranged from 0% (0/14) in Tennessee in 1999 to 26% (14/53) in Georgia in 2001. By using a multivariate logistic regression model and controlling for age and site-to-site variation in prevalence, the proportion of all *Campylobacter* isolates resistant to ciprofloxacin and nalidixic acid in 2001 was significantly higher than the proportion of isolates resistant to ciprofloxacin in 1997 (data only shown for ciprofloxacin in Table 3). The remaining antimicrobial drugs had no statistically significant change in resistance over time (data not shown).

**Table 3:** Trend analysis of the proportion of fluoroquinolone-resistance among *Campylobacter*, National Antimicrobial Resistance Monitoring System, 1997–2001

Y	Unadjusted OR^a^ (95% CI)	Adjusted OR^b^ (95% CI)
1997^c^	1.0	1.0
1998	1.0 (0.6 to 1.7)	1.3 (0.7 to 2.4)
1999	1.4 (0.9 to 2.3)	2.1 (1.2 to 3.9)
2000	1.1 (0.7 to 1.8)	1.5 (0.8 to 2.8)
2001	1.6 (1.0 to 2.5)	2.5 (1.4 to 4.4)

^a^OR, odds ratio; CI, confidence interval. ^b^Adjusted odds ratios were calculated by using logistic regression model, which accounted for site-to-site variation in prevalence. ^c^1997 was the reference value.

Fifty-one percent of *Campylobacter* isolates were resistant to >1 drug, 18% were resistant to >2 drugs, and 10% were resistant to >3 drugs. The most common multidrug resistance (i.e., >2 drugs) pattern included ciprofloxacin, nalidixic acid, and tetracycline.

#### 1997 Retrospective Case-Comparison Study

Sixteen (57%) of 28 ciprofloxacin-resistant *Campylobacter* (CipR) case-patients and 31 ciprofloxacin-sensitive (CipS) case-patients were interviewed. The median age was 46 years (range 9–76 years) for CipR patients and 24 years (range 1–87 years) for CipS patients (Wilcoxon rank-sum, p = 0.08). CipR patients did not differ significantly from CipS patients in terms of sex (40% vs. 42% female, p = 0.1), race (87% vs. 77% white, p = 0.08), and place of residence (87% vs. 61% urban/suburban areas, p = 0.06). Five (31%) CipR patients were hospitalized for gastroenteritis compared with 1 (3%) CipS patient (matched odds ratio [mOR] = 13.6, 95% confidence interval [CI] 1.4 to 130.1). Eight (57%) CipR case-patients reported having bloody diarrhea compared with eight (30%) CipS patients (mOR = 3.2, 95% CI 0.8 to 12.1). Seven (44%) of 16 CipR patients compared with 1 (3%) of 31 CipS patients traveled to a foreign country in the 7 days before illness onset (mOR = 23.3, 95% CI 2.5 to 215.6); 5 (71%) of 7 CipR patients traveled to Europe while 1 CipS patient traveled to the Caribbean. Among all case-patients, 35 of 47 reported treatment with an antimicrobial agent for their illness. Of those who recalled the name of the antimicrobial drug, 75% reported taking a fluoroquinolone, 16% reported taking a macrolide, and 8% took trimethoprim-sulfamethoxazole (TMP/SMX). One CipR patient and one CipS patient took fluoroquinolones between onset of illness and collection of stool specimens. Among the eight CipR patients who did not travel and did not take fluoroquinolones between illness onset and stool specimen collection, seven (87%) consumed poultry in the 7 days before infection; this finding was not statistically different from that in CipS patients. No other exposures were significantly associated with ciprofloxacin-resistant infection, including having pets, drinking raw milk, or being exposed to a farm (data not shown).

#### 1999 Retail Survey

**Figure 1:**
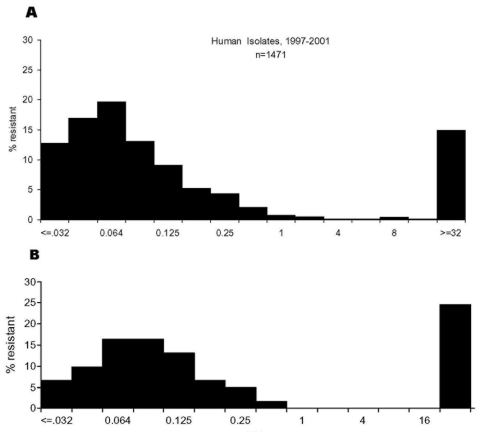
Distribution of ciprofloxacin MICs among *Campylobacter jejuni* isolated from humans and retail chicken. A, human isolates, 1997–2001; N = 1,471. B, grocery store purchased chicken isolates, 1999; N = 62.

Among the 180 retail chicken products purchased, representing 18 domestic brand names from 22 grocery stores, *Campylobacter* was isolated from 80 (44%) samples. Sixty-two (77%) were *C. jejuni*, 16 (20%) were *C. coli*, and 2 (2%) were undetermined (i.e., determination by 16S study did not identify a species). The prevalence of *Campylobacter* isolated was 33% (20 of 60) in Georgia, 37% (22 of 60) in Maryland, and 63% (38 of 60) in Minnesota. This difference among sites was in part due to the difference in isolation rates of *C. coli*; 14 (87%) of the 16 *C. coli* isolates came from retail chickens purchased in Minnesota. A ciprofloxacin-resistant strain of *Campylobacter* was identified in 10% of the 180 retail chicken products tested, and an erythromycin-resistant strain was identified in 2% of chicken products (Table 4). The distribution of ciprofloxacin MICs in *Campylobacter* species of retail chicken and human isolates was similar. For both human and poultry *Campylobacter* isolates, MICs were predominantly <0.5 or >32 µg/mL with few intermediate phenotypes (Figure 1).

**Table 4:** Antimicrobial resistance among *Campylobacter* isolates from retail chicken, by species, National Antimicrobial Resistance Monitoring System, 1999

Antimicrobial agent	% resistant		
	*C. jejuni* (n = 62)	*C. coli* (n = 16)	Other^a^ (n = 2)
Azithromycin	6	0	0
Chloramphenicol	0	0	50
Ciprofloxacin	24	19	50
Clindamycin	5	0	0
Erythromycin	6	0	0
Gentamicin	0	6	0
Nalidixic acid	29	37	50
Tetracycline	69	50	50

^a^One isolate was undetermined (i.e., hippurate-negative *Campylobacter* that could not be further speciated by 16S polymerase chain reaction study), and one isolate was an unknown *Campylobacter* that could not be further characterized.

## Discussion

**Figure 2:**
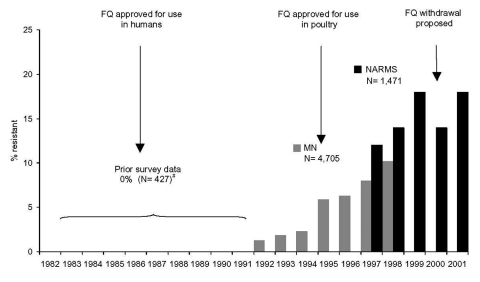
Quinolone- and fluoroquinolone-resistant *Campylobacter jejuni* in the United States, 1982–2001. FQ, fluoroquinolone; MN, Minnesota quinolone resistance among *C. jejuni* strains data (adapted from 18), NARMS, National Antimicrobial Resistance Monitoring System. Prior survey data adapted from reference 19 and 30.

Fluoroquinolone-resistant *Campylobacter* have emerged over the last decade in the United States. In 1990, no ciprofloxacin-resistant human isolates were identified in a national sentinel county-based survey. From 1997 to 2001, the prevalence of ciprofloxacin-resistant *Campylobacter* increased significantly from 13% to 19%. These data are consistent with four prior surveillance studies from humans conducted in the United States: 1) a hospital-based study in Pennsylvania conducted from 1982 to 1991 found no fluoroquinolone resistance among *C. jejuni* isolates (*29*), 2) a second study at the same Pennsylvania hospital found a sharp increase in ciprofloxacin resistance among *C. jejuni* from 8% in 1996 to 40% in 2001 (*30*), 3) a study conducted in Wisconsin between 1992 and 1995 found 12% of the *C. jejuni* to be ciprofloxacin-resistant (*31*), and 4) a study in Minnesota showed an increase in quinolone-resistant *C. jejuni* isolates from 1.3% in 1992 to 10.2% in 1998 (Figure 2) (*18*). The emergence of fluoroquinolone resistance among *Campylobacter* isolates in the 1990s has occurred while resistance to other antimicrobial agents has remained stable. Specifically, resistance to the macrolides, azithromycin and erythromycin, which are commonly used antimicrobial agents in humans (*32*), has remained low (1%–3%).

Our retrospective case-comparison study showed that patients with ciprofloxacin-resistant *C. jejuni* infections were more likely to be hospitalized compared to patients with ciprofloxacin-susceptible infections. These results, however, are based on a small number of patients, and age could have been a confounder. Other studies have found that patients infected with fluoroquinolone-resistant *Campylobacter* have a longer duration of diarrhea than patients with fluoroquinolone-susceptible isolates, although no difference in hospitalization rates have been reported (*18**,**33*). These findings may have clinical implications. Ciprofloxacin is commonly used to treatment severe *Campylobacter* infections and other intestinal infections in adults, so the rise of fluoroquinolone resistance may result in ineffective treatment when fluoroquinolones are used. Macrolides, which are efficacious in treating *Campylobacter* (*5**,**34*), should still be considered the first-line drugs for severe *Campylobacter* infections, as resistance to this class remains low.

Our study also identified foreign travel, particularly to Europe, to be associated with ciprofloxacin-resistant *C. jejuni* infection. High rates of fluoroquinolone-resistant *Campylobacter* have been reported from southern Europe and other regions of the world (*13*). Studies in northern Europe have associated fluoroquinolone use in food animals, particularly poultry, as a source for human infection with fluoroquinolone-resistant *Campylobacter* (*13*). Nevertheless, while foreign travel was a risk factor in our study, over half of the ciprofloxacin-resistant infections were domestically acquired. Ciprofloxacin resistance was not associated with use of fluoroquinolones before specimen collection, which suggests that fluoroquinolone-resistant organisms did not result from individual use of fluoroquinolones. A more recent, larger case-control study of patients infected with ciprofloxacin-resistant *Campylobacter* infections found similar results to our study; 58% of illnesses were domestically acquired, and none of the patients took fluoroquinolones after illness onset and before specimen collection (*35*).

Our 1999 survey of retail chicken sold in selected supermarkets provided ecologic evidence that chicken may be a source of domestically acquired ciprofloxacin-resistant *Campylobacter* infections; 10% of retail chickens were contaminated with ciprofloxacin-resistant *Campylobacter*. Other studies have shown that *Campylobacter*, including ciprofloxacin-resistant *Campylobacter*, are commonly isolated from retail poultry meats. A survey of retail meats purchased in the Washington, D.C., area isolated *Campylobacter* species from 71% of chicken and 14% of turkeys tested (*36*); 25% of the *C. jejuni* isolates and 40% of the *C. coli* isolates were resistant to ciprofloxacin (*37*). A second survey in Minnesota isolated *Campylobacter* from 88% of retail chicken meats purchased in 1997, including *C. jejuni* in 74% and *C. coli* in 21%. Ciprofloxacin-resistant *Campylobacter* was identified in 20% of retail chicken products (*18*). In this study, comparison of molecular subtypes from human and retail chicken quinolone-resistant *C. jejuni* isolates found that six of seven subtypes were indistinguishable from each other.

In the United States, the FDA has approved the use of fluoroquinolones at different times for humans and food animals. Fluoroquinolones have been commonly used in humans for treating intestinal and other infections since 1986 (*32*). The first fluoroquinolones to be FDA-approved for use in food animals in the United States were sarafloxacin in 1995 and enrofloxacin in 1996. These fluoroquinolones were approved for use in chickens and turkeys to treat bacterial respiratory infections principally caused by *E. coli*. These agents are typically administered to the entire poultry house (often >20,000 birds) through drinking water, which results in the treatment of sick and healthy birds with various concentrations of fluoroquinolones. The extent of fluoroquinolone use in chickens and turkeys in the United States is not known; manufacturers and farmers are not required to report these data. The Animal Health Institute has estimated that 1%–2% of the approximately 8 billion broiler chickens slaughtered each year in the United States are treated with fluoroquinolones (*38*). An experiment with *Campylobacter*-infected chickens treated with enrofloxacin and sarafloxacin showed that ciprofloxacin resistance rapidly developed among *Campylobacter* (*39*).

An association between the approval of fluoroquinolones for use in food-producing animals and the development of fluoroquinolone-resistant *Campylobacter* in animals and humans has been noted in several countries. The approval of fluoroquinolones for use in food animals has been followed temporally by a rise in ciprofloxacin-resistant *Campylobacter* and other enteric pathogens isolated from animals and humans in Denmark, the Netherlands, and Spain (*13**,**40*). After the use of oral fluoroquinolones in pigs was discontinued in Denmark in 1999, nalidixic acid resistance among *C. coli* isolates from pigs decreased from 17% in 1998 to 5% in 2001 (*41*). In the United States, FDA has recently conducted a quantitative risk assessment and concluded that fluoroquinolone use in chickens and turkeys results each year in >10,000 human infections with fluoroquinolone-resistant *Campylobacter* in persons who seek medical care and are treated with fluoroquinolones (*42*). FDA proposed the withdrawal of approval of fluoroquinolones for use in poultry in October 2000 (*43*). This is the first time a proposal has been made to withdraw an approval for an antimicrobial used in agriculture because of associated emergence of resistance in humans. The manufacturer of sarafloxacin has since withdrawn this product from the market, but the manufacturer of enrofloxacin continues to market enrofloxacin for use in poultry in the United States.

Our studies had several limitations. The retrospective case-comparison study did not assess exposures among travelers and therefore cannot assess the possibility that the travelers may have acquired ciprofloxacin-resistant *Campylobacter* from eating poultry or other foods while traveling. Routine surveillance for antimicrobial susceptibility among *Campylobacter* did not start until 1997, and therefore we cannot identify national trends in antimicrobial resistance from 1991 to 1996. Other limitations are evident in NARMS *Campylobacter* surveillance, including the use of sentinel clinical laboratories in some states and some variation in the isolation procedures. However, these limitations are not likely to be associated with an increased (or decreased) likelihood of selecting antimicrobial-resistant isolates for submission to NARMS since the antimicrobial resistance pattern of the isolates were not known when the isolates were selected. Lastly, because NARMS *Campylobacter* surveillance was not nationwide and resistance may differ regionally, generalization to the U.S. population should be done with caution.

In summary, we describe the emergence over the last decade of fluoroquinolone-resistant *Campylobacter* infections in the United States. As of 1997, more than half of such infections were domestically acquired. In 1999, fluoroquinolone-resistant *Campylobacter* organisms were present on a substantial fraction of chickens sold at supermarkets in three widely separated locations in the United States. Continuing national surveillance of human infections and prospective national monitoring of the frequency of contamination of poultry at retail would provide useful ongoing information. Clinicians should include macrolides, such as azithromycin, as a first-line treatment of severe *Campylobacter* infections.

## Footnotes

1 This isolate was reported to be ciprofloxacin resistant in reference 20; more recently established interpretive criteria define it as susceptible.
